# Development and Implementation of a Cloud-Based Meningitis Surveillance and Specimen Tracking System in Burkina Faso, 2018

**DOI:** 10.1093/infdis/jiz376

**Published:** 2019-10-31

**Authors:** Alpha Oumar Diallo, Tanga Kiemtoré, Brice Wilfried Bicaba, Isaïe Medah, Tiga Félix Tarbangdo, Simon Sanou, Heidi M Soeters, Ryan T Novak, Honoré Flavien Aké

**Affiliations:** 1 National Center for Immunization and Respiratory Diseases, Centers for Disease Control and Prevention, Atlanta, Georgia; 2 Ministère de la Santé, Ouagadougou, Burkina Faso; 3 Davycas International, Ouagadougou, Burkina Faso

**Keywords:** Burkina Faso, Africa, meningitis, cloud-based health information system, specimen tracking system, integrated disease surveillance

## Abstract

Nationwide case-based meningitis surveillance was established in Burkina Faso following the introduction of meningococcal serogroup A conjugate vaccine in 2010. However, timely tracking and arrival of cerebrospinal fluid specimens for confirmation at national reference laboratories remained suboptimal. To better understand this gap and identify bottlenecks, the Burkina Faso Ministry of Health, along with key partners, developed and implemented a cloud-based System for Tracking Epidemiological Data and Laboratory Specimens (STELAB), allowing for timely nationwide data reporting and specimen tracking using barcodes. STELAB was adapted to Burkina Faso’s infrastructure to ensure suitability, functionality, flexibility, and sustainability. We describe the design, development, and implementation of STELAB. In addition, we discuss strategies used to promote sustainability, lessons learned during the first year of implementation, and future directions. STELAB’s novel design and country-driven approach has the potential to achieve sustainable real-time data reporting and specimen tracking for the first time in sub-Saharan Africa.

Burkina Faso, a West African country with 19 million people, experiences hyperendemic meningitis and is at elevated risk of recurrent meningitis outbreaks. Consequently, the Burkina Faso government is committed to controlling meningitis, and vaccinated 96% of the target population aged 1–29 years with the meningococcal serogroup A conjugate vaccine (MACV; MenAfriVac) via a mass vaccination campaign in 2010 [1]. The successful MACV campaign nearly eliminated disease due to *Neisseria meningitidis* serogroup A (NmA) [1]. Following the detection and response to 5 NmA cases during the 2014–2015 meningitis season [2], Burkina Faso conducted a MACV catch-up campaign for children aged 1–6 years in 2016 and introduced MACV into the routine childhood vaccination program in 2017 [2].

The World Health Organization (WHO) recommended that each country that introduced MACV should conduct case-based meningitis surveillance (CBS) to monitor the effectiveness of the vaccine and disease trends as well as to help with outbreak response [3]. Following a CBS pilot [1], the Burkina Faso Ministry of Health (MoH) expanded CBS nationwide in 2010 after the MACV mass campaign, thereby increasing the proportion of suspected meningitis cases with a cerebrospinal fluid (CSF) specimen collected and the identification of bacterial meningitis pathogens [1, 2].

Improvements in CBS were supported by Burkina Faso’s membership in the MenAfriNet Consortium since 2014. MenAfriNet supports the strategic implementation of CBS in the meningitis belt and is an international consortium led and implemented by African Ministries of Health, Agence de Médecine Préventive, the US Centers for Disease Control and Prevention (CDC), and WHO, with support and collaboration from other international and nongovernmental organizations [4]. Burkina Faso is a key country for CBS because it has one of the highest meningitis disease burdens globally, was one of the first countries to introduce MACV nationally via a mass campaign, and has a history of successful public health leadership and partnership [2, 5, 6].

Despite these successes, meningitis surveillance challenges remained, especially regarding timely transport and tracking of CSF specimens sent to a national reference laboratory (NRL) for confirmation via culture or real-time polymerase chain reaction (rt-PCR). Prompt detection of suspected case clusters and timely identification of causative serogroups are vital functions of meningitis surveillance. To monitor the surveillance system’s ability to perform these functions, surveillance performance indicators are assessed quarterly [7]. Through these routine analyses and assessments, the country recognized long delays between CSF specimen collection and arrival at 1 of the 5 NRLs. Although a high proportion of suspected meningitis cases had CSF specimens collected (98% in 2016) and 86% of CSF specimens were arriving at an NRL, timeliness was suboptimal, especially during the epidemic season (December to June). For example, although WHO CBS guidelines recommend that ≥50% of CSF specimens should arrive at an NRL within 7 days of collection [3], only 16% of CSF specimens in Burkina Faso met this timeliness threshold in 2016 (unpublished data).

As one of the first steps to addressing this gap, Burkina Faso sought to identify bottlenecks in the specimen transport system and improve efficiency. However, there was no available system to track CSF specimens in real-time from the time of collection through the district and regional laboratory network and finally to an NRL for pathogen confirmation. Therefore, in 2016–2018, the Burkina Faso MoH, in collaboration with Davycas International (a local nongovernmental organization) and CDC, developed and implemented a cloud-based software system, System for Tracking Epidemiological data and Laboratory Specimens (STELAB), allowing for timely nationwide data reporting and specimen tracking using barcodes. We describe the context for STELAB and its role in meningitis surveillance in Burkina Faso, as well as its design, development, deployment, and evolution. In addition, we discuss strategies used to promote the sustainability of the system, lessons learned during the first year of implementation, and future directions.

## MENINGITIS SURVEILLANCE IN BURKINA FASO

In Burkina Faso, there are 2 complementary systems of nationwide population-based meningitis surveillance, which have been previously described [2]. In brief, the aggregate surveillance system collects weekly reports of suspected meningitis cases, defined as a sudden onset of fever ≥38.5°C with neck stiffness, altered consciousness, or other meningeal signs (including flaccid neck, bulging fontanelle, or convulsions in young children) [8], and deaths from both inpatient and outpatient facilities aggregated at the district level. This system contains no identifying patient information or laboratory data. A complementary system, nationwide CBS, collects case-level demographic and clinical information as well as results of CSF examination and laboratory testing using Integrated Disease Surveillance and Response (IDSR) tools [9]. A paper case notification form is completed for each suspected meningitis case identified at peripheral health posts, district, regional, or teaching hospitals. A unique case identification number (EPID number) is assigned by the district surveillance officer. If a CSF specimen is collected, a copy of the form with the assigned EPID number travels with the specimen to a laboratory. In Burkina Faso, meningitis CBS is unique because specimens can be tested at all 3 levels of the public health system (district, regional, and national), in contrast to surveillance for diseases like measles and yellow fever, where all testing is conducted solely at a designated reference laboratory. Additionally, specimens with a negative test result at a lower level can be tested at a higher level. District laboratories (n = 82) have the capacity to conduct macroscopy, cytology, Gram stain, and latex agglutination; regional laboratories (n = 9) have the same capacity as district laboratories in addition to culture capacity; and the NRLs (n = 5) have the same capacity as the regional laboratories in addition to rt-PCR capacity. Three NRLs are located in Ouagadougou, the capital, and 2 are in Bobo-Dioulasso, the second largest city; these locations minimize specimen transport time and distance and ensure NRL capacity throughout the country.

A CSF specimen transport mechanism exists and is funded by the Burkina Faso MoH and CDC. Generally, health workers personally transport CSF specimens collected at peripheral health posts to the nearest laboratory (district, region, or NRL) within 24 hours via public transport or motorcycle, whereas all other health facilities have a laboratory on-site. CSFs first received at a district or regional laboratory are tested and then batched and transported personally by a microbiologist to an NRL via the district service vehicle or public transportation. The frequency of transport varies depending on availability of personnel, district service vehicle, and resources. Once laboratory results are available, surveillance officers and laboratory data managers at each level of the public health system enter laboratory results and clinical and demographic data for all cases, including for cases with no available CSF specimen, into a database. Data are reported to the NRLs and the Directorate for Disease Control (DDC) weekly, including zero reporting. DDC merges and shares the complete data with districts and regions to update their own databases. Districts feedback test results to peripheral health posts.

## STELAB OVERVIEW

### Design and Development

STELAB, developed by Davycas International and financed by the MenAfriNet Consortium, is a hybrid—online and offline—application with a web interface for data entry, validation, reporting, and visualization developed using the latest web technology (PHP 5.6, Bootstrap CSS3, HTML5, JavaScript) and MongoDB, a NoSQL (Big Data) database compatible with MySQL. To encourage use of the system in Burkina Faso, where internet access is not readily available, the application was made installable on computers, allowing users to enter, validate, analyze, and visualize data already saved on their computers while offline. The installed application automatically pulls new version updates when online. A built-in program routinely verifies the availability and quality of the mobile broadband network to transfer data to or receive data from the cloud server. The cloud server pushes data to the Directorate of Informatics and Telemedicine’s (DIT) local server via a satellite communication system. DIT is connected to DDC on an MoH intranet via a fiber-optic cable.

**Figure 1.**
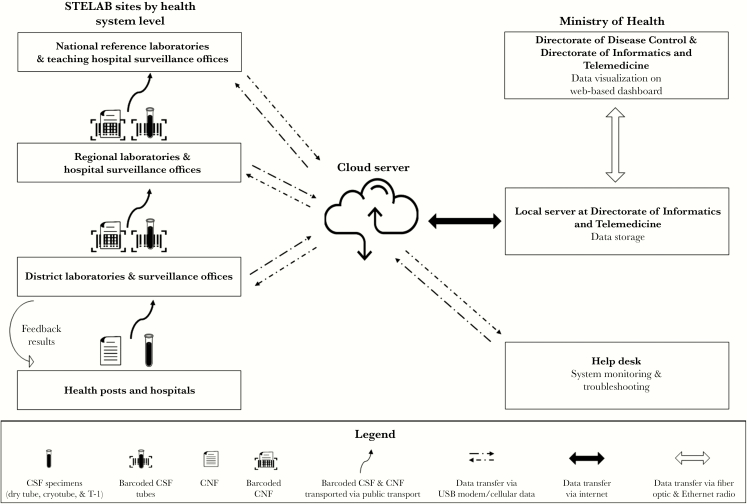
Data and specimen flowchart and representation of System for Tracking Epidemiological Data and Laboratory Specimens tools available at each site. Abbreviations: CNF, case notification form; CSF, cerebrospinal fluid; STELAB, System for Tracking Epidemiological Data and Laboratory Specimens; T-I, trans-isolate medium; USB, Universal Serial Bus.

The DDC prints and distributes barcode labels to districts at the beginning of the year based on historical average number of cases reported, monitors their use via STELAB, and sends new labels as needed to prevent stockouts. Each time a suspected meningitis case is identified and a CSF specimen is collected, the specimen is sent to a district laboratory with the corresponding case notification form. Four labels with the same barcode are printed for each case. One label is placed on the case notification form and another on the clear tube containing the CSF specimen (Figure 1). The CSF specimen is aliquoted into a cryotube for rt-PCR testing and into trans-isolate media for culture; the remaining 2 labels are placed on these tubes.

The barcode is scanned into the STELAB system, which then tracks the specimen’s journey from the district laboratory to the regional laboratory and finally to an NRL. In addition to tracking when specimens are received and transported to the next level of the laboratory pyramid, microbiologists also enter laboratory test results into the system. The STELAB system assigns the EPID number based on the unique barcode, thereby avoiding any errors or duplication of EPID numbers that occurred when district surveillance officers assigned numbers manually [10]. The DDC, DIT, laboratories, and other involved MoH departments can monitor the progress of each specimen via a web-based dashboard.

### Implementation: Pilot, Deployment, and Evolution

STELAB was piloted in 15 district laboratories, 7 regional laboratories, and 4 NRLs from February to April 2017. Twenty-eight microbiologists participated in a 2-day training consisting of both lectures and hands-on practice in Ouagadougou. User profiles were set up according to job roles (Table 1), and sites were provided a set of equipment and materials according to their function (Table 2). Following installation, users began to use STELAB.

**Table 1.** Type and Number of Sites Using System for Tracking Epidemiological Data and Laboratory Specimens (STELAB) and Number of Users Trained

Level	STELAB Site^a^	No. of Sites^b^	STELAB Users	No. of Users Trained^c^
National	Teaching hospital surveillance office	3	Hospital surveillance officers and data managers	11
	National level laboratory	5	Microbiologists	31
	Directorate of Disease Control	1	Lead data managers	4
		1	Laboratory point of contact	1
	Directorate of Informatics and Telemedicine (houses server room)	1	Information technology technicians	6
Regional	Hospital surveillance office	9	Regional hospital surveillance officers	41
	Hospital laboratory	9	Microbiologists	87
District	Surveillance office	70	District surveillance officers	122
	Hospital laboratory	82	Microbiologists	258
Total		181		561

Abbreviation: STELAB, System for Tracking Epidemiological Data and Laboratory Specimens.

^a^French terms for surveillance sites: teaching hospital surveillance office (Centre hospitalier universitaire, services de planification et d’information hospitalière/services d’information médicale); national-level laboratory (Laboratoire du niveau national); Directorate of Disease Control (Direction de la Promotion de la Santé de la Population); Directorate of Informatics and Telemedicine (Direction des Services Informatiques et de la Télésanté); regional hospital surveillance office (Centre hospitalier régional, services de planification et d’information hospitalière/services d’information médicale); regional hospital laboratory (Centre hospitalier régional); district surveillance office (Centres d’information sanitaire et de surveillance épidémiologique); district hospital laboratory (Centre médical/centre médical avec antenne chirurgicale).

^b^All sites received on-site training and the necessary materials listed in Table 2. Eight districts have >1 district-level laboratory.

^c^Although chiefs of national reference laboratories (n = 5) and chief district medical officers (n = 67) are not primary users of STELAB, they were also trained on STELAB to encourage its use and promote analysis of data for decision making.

**Table 2.** Type and Quantity of Equipment Distributed by Site

Site	Equipment	Quantity
National-level/reference laboratories, hospital laboratories, and surveillance offices	Desktop computer	181
	Uninterrupted power supply and voltage regulators	181
	Barcode scanner	181
	Internet connection, USB modem	181
	SIM card	181
Directorate of Disease Control	Desktop computer	1
	Laptop computer	3
	Large visualization monitor	3
	Barcode printer	2
Directorate of Informatics and Telemedicine	Large visualization monitor	1
	Server with accessories	1
	Uninterrupted power supply	1
	Electricity generator	1
Help desk (stocks equipment reserved for maintenance)	Desktop computer	6
	Uninterrupted power supply and voltage regulators	32
	Barcode scanner	10
	Barcode printer	1
	Internet connection USB modem	10
	SIM card	10

Abbreviations: SIM, subscriber identification module; USB, Universal Serial Bus.

Each site was asked to enter real meningitis case records, or fictitious records when cases were not available, to test the functionality of the system including internet availability. A team of staff from DIT and Davycas International monitored the functionality of STELAB, collected feedback on the system design, and responded to inquiries via phone. When they could not resolve problems by phone, a technician was sent to the site. The STELAB development team resolved bugs and implemented relevant design and functionality feedback for the next version.

**Figure 2.**
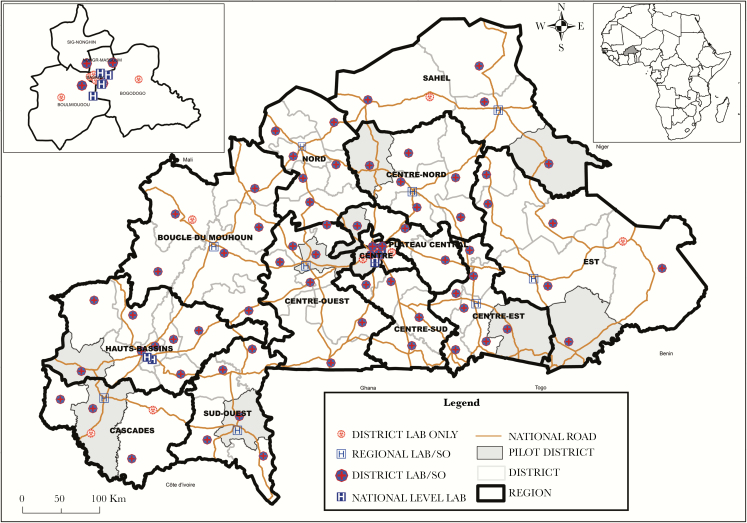
Map of System for Tracking Epidemiological Data and Laboratory Specimens (STELAB) laboratories, surveillance offices, and implementation phases. The teaching hospital surveillance offices and 3 of the 5 national level laboratories are located on teaching hospital campuses in Ouagadougou (Centre Hospitalier Universitaire-Yalgado Ouédraogo and Centre Hospitalier Universitaire Pédiatrique Charles de Gaulle) and in Bobo-Dioulasso (Centre Hospitalier Universitaire Souro Sanou). The two remaining national level laboratories are located at the national public health laboratory, (Laboratoire National de la Santé Public, Ouagadougou), and at the research center, Centre Muraz, in Bobo-Dioulasso. Centre Hospitalier Universitaire Pédiatrique Charles de Gaulle serves as the designated national meningitis reference laboratory. All 9 regional hospital laboratories and hospital surveillance offices are located on the regional hospital campuses. All 82 district-level laboratories and 61 of 70 district surveillance offices are located on district hospital campuses. The remaining 9 district surveillance offices are located in districts serving as regional capitals and are located separately from the district hospital. Abbreviation: SO, surveillance office.

Following the successful pilot phase, STELAB was scaled up to provide nationwide coverage in June 2017 (Figure 2). STELAB was implemented at the district (152 sites), regional (18 sites), and national (11 sites) levels consisting of surveillance offices and laboratories. In particular, all of the public laboratories that conduct meningitis testing (n = 96) were included. Due to limited infrastructure, STELAB was not implemented in peripheral health posts at this time.

In September 2017, DDC, CDC, and USAID met to review STELAB and results from the national scale-up, to explore other opportunities to strengthen the surveillance system and to define the next steps. One of the critical decisions made in this meeting was to extend STELAB from a specimen-tracking system with laboratory test results to a system that also collects information on patient demographic and clinical information. This meant that both microbiologists and surveillance officers at all levels of the surveillance system would have access to the same STELAB system.

From November to December 2017, trainings were conducted in each district (n = 70), regional hospital (n = 9), NRL (n = 5), and directorate (n = 2), including all microbiologists and surveillance officers who would potentially enter data in STELAB (Table 1). During trainings, equipment was deployed and installed at all sites (n = 181), including laboratories and surveillance offices (Table 2). Prior to trainings, outreach efforts and meetings with district medical officers and regional health directors were organized to ensure high-level commitment to this strategy.

**Figure 3.**
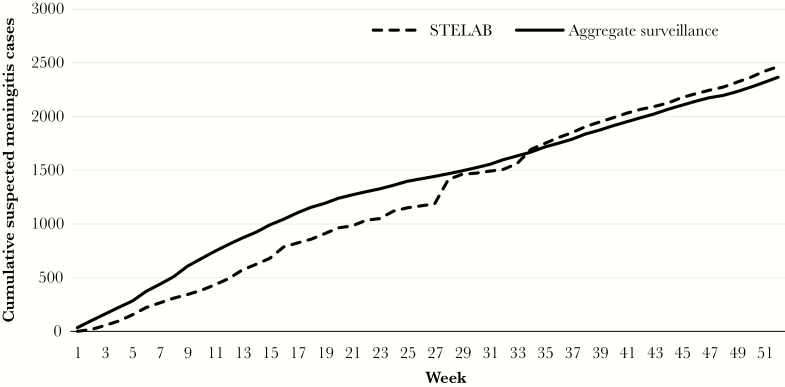
Cumulative meningitis cases reported in System for Tracking Epidemiological Data and Laboratory Specimens (STELAB) compared to aggregate national meningitis surveillance, Burkina Faso, 2018. Comparisons of data from all 70 districts in 2018 show that over time, the cumulative number of meningitis cases reported in STELAB surpassed those reported in the aggregate national meningitis surveillance as users became familiar with STELAB. Additionally, various technical and training issues in 2 large districts reporting many meningitis cases were resolved during week 27, thereby increasing the STELAB case counts to the level reported in aggregate surveillance.

STELAB officially went live nationally on 1 January 2018. The system is being used in parallel with the IDSR linelist, which also collects minimal case level information as part of aggregate surveillance, to compare the performance of the 2 systems. Once the results from this comparison demonstrate that STELAB is consistently comparable or better with regard to data completeness and quality, then DDC will halt the double data entry and maintain only the STELAB system. Preliminary comparisons of data from 2018 show that, over time, the cumulative number of meningitis cases reported in STELAB surpassed those reported via aggregate meningitis surveillance as it improved and users became familiar with STELAB (Figure 3). For example, weak internet signals at laboratories and surveillance offices of 2 large districts reporting many meningitis cases and the need for refresher trainings on how to use the system prevented the transfer of data to the server. A technical team provided an amplification antenna and an on-site refresher training, which increased the STELAB case count to the level reported in aggregate surveillance in week 27 (Figure 3). STELAB will be evaluated in 2019–2020 to determine its impact on improving timeliness of data reporting quality and to inform modifications of data reporting and specimen transport to guide rapid response to outbreaks.

### Data Security and Sharing

The DDC controls access to data on stored on STELAB, which is password protected and regularly updated with security patches and relies on data encryption provided through MongoDB. Users can only view case records that fall within their jurisdictions. To ensure data integrity, data editing rights are controlled by the national surveillance office. The DDC shares de-identified data with partners, including WHO and CDC.

## STELAB: LESSONS LEARNED

### Ownership, Sustainability, and Integration

Sustainability served as the guiding principle of the design and implementation of STELAB. The system was designed and led by a collaboration of key directorates within the Burkina Faso MoH, resulting in clear ownership and prioritization, and therefore greater sustainability. This effort has also encouraged more collaboration and communication between these directorates. Additionally, the decentralized site-based training approach maximized the number of users trained directly by the implementation team and thus promoted the ownership and sustainability of the system.

The features of the STELAB application and implementation strategy have promoted its use and timely data reporting and specimen tracking, and integrated STELAB within the broader public health infrastructure. STELAB was designed specifically for Burkina Faso’s infrastructural context, where internet access is not readily available. During laboratory visits prior to STELAB deployment, it was observed that many laboratories have computers, but they are not used on a daily basis as they are allocated to a specific program or in need of repair. To ensure that the STELAB equipment is regularly used to report meningitis cases and also contributes to the daily functions of microbiologists and surveillance officers, Microsoft Office and antivirus software were included. In addition to transmitting CBS data to the server via STELAB, district surveillance officers have been using the provided mobile broadband network to submit monthly aggregated data reports from district surveillance offices to the regional surveillance office or DDC.

Data elements for cholera, dengue, measles, shigellosis, viral hemorrhagic fevers, measles, severe acute respiratory infections, and yellow fever surveillance have been harmonized and integrated into STELAB. Nationwide surveillance reporting and specimen tracking for these diseases in STELAB will begin in 2020. Polio has not been integrated into STELAB at this time because it has functional systems in place, and polio uses a different specimen referral system. STELAB also has the capacity to include diseases that do not require laboratory confirmation.

### Surveillance System Optimization

Timely reporting and visualization of data at all levels of the health system including peripheral health facilities has allowed surveillance officers at the national level to correct data reporting errors immediately, rather than wait for quarterly or biannual data validation exercises. In contrast to aggregate surveillance data, which are transmitted weekly from district and regional surveillance offices to DDC but with a 1-week lag, STELAB case-based data, including laboratory results, are transmitted to the server almost immediately after they have been entered, thus giving DDC a timely understanding of the national epidemiologic situation.

STELAB has also optimized suspected case notification while fostering collaboration between key actors, microbiologists, and surveillance officers at all levels of the surveillance system. For example, microbiologists are now able to report suspected cases to the surveillance system, in addition to reporting done by clinicians. Communication between district microbiologists and district surveillances officers has also increased because they share the same system and collaborate to resolve data entry discrepancies.

### Installation of a Help Desk

It quickly became apparent that a help desk was needed to monitor the functionality of the system, resolve technical issues, and gather feedback. The help desk addresses difficulties including limited computer literacy, barcode label stockouts, software bugs, and challenges with power supply or internet access. The help desk, manned by the MoH and Davycas International staff, resolves most of these challenges remotely via telephone. For broken equipment, they quickly dispatch replacements. Upon notification of a bug in the software, the help desk informs the development team, who quickly resolve the bug and push updated software to the sites via the cloud. Some sites have also required installation of a signal amplification system to improve internet access. The help desk also tracks a set of weekly indicators to monitor system performance and inform improvements. These include the number of cases and deaths reported by the aggregated surveillance compared to those recorded in the STELAB database by district and week, the number of CSF specimens with a final NRL result listed on the DDC bulletin compared to those with a final result in the STELAB database, and the number of districts that have not interacted (eg, no data entry, no internet connection) with the STELAB server during the previous 2 weeks. The help desk is now an integral part of the Burkina Faso Emergency Operations Center (EOC).

### Cost and Funding Implications

MenAfriNet funded the development, implementation, and maintenance of STELAB with contributions from the Burkina Faso MoH. An evaluation of the implementation of STELAB, funded by WHO-Geneva, to inform the feasibility of implementing STELAB in other meningitis belt countries documented that it cost about US$1 035 000 to develop STELAB (hiring developers, cloud services, etc), purchase equipment (Table 2), train users (pilot and site-based trainings), and operate STELAB for the first year in Burkina Faso [11]. The annual STELAB operational cost, which includes internet subscriptions, equipment maintenance, help desk staffing and refresher trainings, is estimated at US$ 245 000. Since January 2019, the operational expenses have been included in the DDC surveillance unit and EOC annual budget. It will take 2–3 years of inclusion in the MoH budget for the operational expenses to be financed by the Burkina Faso government. As with any new technological system, initial development and implementation (materials and trainings) are expensive. However, expenses decrease with time, and integration of other epidemic-prone diseases under case-based surveillance into STELAB will further economize resources [12].

## DISCUSSION

Timely case confirmation and reporting are critical to inform rapid outbreak response and monitor disease trends. Although CSF specimen collection for suspected meningitis cases improved in Burkina Faso following the introduction of MACV in 2010, timely arrival of CSF specimens at NRLs for confirmation via culture and rt-PCR remained suboptimal. To better understand the gaps and identify bottlenecks in the CSF specimen transport system, the Burkina Faso MoH, along with key partners, designed and implemented a cloud-based meningitis surveillance and specimen tracking system, STELAB. The hybrid—online and offline—and flexible design of the STELAB application was adapted to the Burkina Faso infrastructure to ensure suitability, functionality, and sustainability.

Because STELAB was built on the IDSR framework and interoperates with other health information systems used by the MoH, such as District Health Information System (DHIS2) and other mobile health systems, STELAB can serve as a platform for timely data reporting and specimen tracking for other epidemic-prone diseases under case-based surveillance. As most of these diseases are preventable by vaccination, the data collected via this platform could also inform evaluations of the effectiveness of national vaccination programs. Consequently, the MoH integrated anthrax, dengue, brucellosis, cholera, rabies, severe acute respiratory infections, and shigellosis surveillance into STELAB in 2019. An interoperability system is in development to aggregate case-based data from STELAB and automate data transfer to DHIS2, which is used as an aggregated data storage system.

The MoH and its partners will conduct an evaluation of STELAB in 2019, after the first full year of data collection, to determine the impact of the implementation of this system on the completeness and quality of data as well as the timeliness of data reporting, specimen arrival to the NRL, and reporting of test results. The MoH is also taking measures to reduce operating costs and promote the sustainability of the STELAB platform, such as working with mobile broadband internet providers to reduce internet cost and providing ongoing trainings to address staff turnover. DIT is also integrating STELAB sites into the government intranet to serve as a backup system for data sharing.

Implementation of STELAB has connected the national network of laboratories and surveillance offices electronically for the first time, allowing for timely tracking of specimen transport and pathogen confirmation to monitor disease trends and evaluate the effectiveness of national vaccination programs. STELAB’s novel design and country-driven approach has the potential to achieve sustainable real-time data reporting and specimen tracking for the first time in sub-Saharan Africa.

## Notes

***Acknowledgments.*** The MenAfriNet Consortium (www.menafrinet.org) is an international consortium led and implemented by the Ministère de la Santé du Burkina Faso, Ministère de la Santé et de l’Hygiène Publique du Mali, Ministère de la Santé Publique du Niger, Ministère de la Santé Publique du Tchad, Ministère de la Santé et de la Protection Sociale du Togo, Agence de Médecine Préventive, US Centers for Disease Control and Prevention (CDC), and the World Health Organization (WHO), with support and collaboration from other international and nongovernmental organizations. The authors thank all MenAfriNet partners, including participating national health systems, health centers, and laboratories.

***Financial support.*** This work was funded by the MenAfriNet consortium (www.menafrinet.org) through a grant from the Bill & Melinda Gates Foundation (OPP1084298).

***Supplement sponsorship. ***This work is part of a supplement sponsored by MenAfriNet Consortium.

***Potential conflicts of interest.*** The authors: No reported conflicts of interest. All authors have submitted the ICMJE Form for Disclosure of Potential Conflicts of Interest.
